# Appearance-Based Salient Regions Detection Using Side-Specific Dictionaries

**DOI:** 10.3390/s19020421

**Published:** 2019-01-21

**Authors:** Mian Muhammad Sadiq Fareed, Qi Chun, Gulnaz Ahmed, Adil Murtaza, Muhammad Rizwan Asif, Muhammad Zeeshan Fareed

**Affiliations:** 1 School of Electronic and Information Engineering, Xi’an Jiaotong University, Xi’an 710049, China; sadiqfareed@mail.xjtu.edu.cn (M.M.S.F.); rizwanasif@ciitlahore.edu.pk (M.R.A.); 2 School of Management, Xi’an Jiaotong University, Xi’an 710049, China; zeeshan.fareed@ist.edu.pk; 3 School of Science, MOE Key Laboratory for Non-equilibrium Synthesis and Modulation of Condensed Matter, State Key Laboratory for Mechanical Behaviour of Materials, Xi’an Jiaotong University, Xi’an 710049, China

**Keywords:** salient region detection, appearance based model, regression based model, human visual attention, background dictionary

## Abstract

Image saliency detection is a very helpful step in many computer vision-based smart systems to reduce the computational complexity by only focusing on the salient parts of the image. Currently, the image saliency is detected through representation-based generative schemes, as these schemes are helpful for extracting the concise representations of the stimuli and to capture the high-level semantics in visual information with a small number of active coefficients. In this paper, we propose a novel framework for salient region detection that uses appearance-based and regression-based schemes. The framework segments the image and forms reconstructive dictionaries from four sides of the image. These side-specific dictionaries are further utilized to obtain the saliency maps of the sides. A unified version of these maps is subsequently employed by a representation-based model to obtain a contrast-based salient region map. The map is used to obtain two regression-based maps with LAB and RGB color features that are unified through the optimization-based method to achieve the final saliency map. Furthermore, the side-specific reconstructive dictionaries are extracted from the boundary and the background pixels, which are enriched with geometrical and visual information. The approach has been thoroughly evaluated on five datasets and compared with the seven most recent approaches. The simulation results reveal that our model performs favorably in comparison with the current saliency detection schemes.

## 1. Introduction

Salient Region Detection (SRD) is a procedure to confine the image according to human visual attention and discovers the most useful and informative portion of an image. This procedure tries to approximate the possibility that the image region that is taking more attention comes out as a salient object. It is also a very helpful step because it is applied in many computer vision applications to reduce the computational complexity by only focusing on the salient parts of the image. The conventional saliency methods are separated into two groups as the bottom-up [1] and top-down [2]. The first category is a bottom-up method, which is a stimuli-driven approach and it only depends on the prior knowledge of the object and the background. Whereas, the second category is a top-down approach, which is data-driven and does not need prior information to detect the saliency.

The major portion of SRD literature [3,4,5] is comprised of the bottom-up approaches [1], as these methods only consider low-level features and demonstrate a remarkable performance. The dense and sparse appearance-based models are separately applied in [6,7] for the salient region computation. The dense reconstruction error-based methods [8] have persuasive results when the image border is large and contains the sparsely connected regions. However, these methods lose their efficiency when the background contains a latent pattern or the background is complicated with small-scale high-contrast patterns. The dense appearance-based models [7] provide a more expressive and generic description of the background. These methods are more sensitive towards the background noise. So, the dense representation error-based models are very less useful in detecting the salient objects with a cluttered background. The methods based on a background template set [9,10,11], and co-similarity matrix [7] have convincing results whenever the salient objects pop out closer to the center part of the scene. However, when the salient objects significantly touch the image boundary, parts of them are wrongly considered as background. Consequently, the extracted saliency is less accurate when the salient object part is popping out or touching the boundary. In this case, the foreground parts of the image are mistakenly considered as the reconstructive dictionary and obtain zero weights, and the salient objects in the remaining parts of the image are found to be less accurate.

In this paper, we introduce a novel SRD method which fuses the compact appearance and discrimination of the individual scenes into a combined framework. Firstly, the input images are segmented into superpixels. Secondly, we employ the appearance-based model to measure the rareness of the features. Thirdly, we apply the regression-based model to rank the previously computed results on the basis of the foreground and the background multi-feature cues, respectively. Finally, we utilize an optimization method to produce an even and accurate salient region map. Our appearance-based model is very simple and easily detects the objects closer to the boundary of the scene. Our regression-based model makes the initial saliency map smoother and it is very helpful in highlighting the salient object part. The proposed method utilizes the visual, geometrical and location information for SRD and shows improved results as compared to the previous contrast-based methods. To fuse the previously obtained results, we applied an enhancement procedure to compute more even and precise salient region maps. We compare our method visually as well as graphically against the seven current SRD methods on the five benchmark databases. From the qualitative and quantitative evaluation, we found that our method performance remains very consistent on all the selected databases. The main contributions of our method are summarized as follows:

- The designed model is robust and easily handles the cluttered and noisy background which was a problem for dense appearance-based models. Also, the side-specific dictionaries of the proposed model are helpful in detecting the salient objects adjacent to the boundary.
- Sometimes the small segments from the background are extremely highlighted and affect the computed saliency. The averaging process of the proposed model is very helpful to overcome this issue by measuring the saliency of a superpixel as an average residual in this segment.
- To enhance the discrimination between the foreground and the background, we engage a multi-feature graph-learning procedure which incorporates the intrinsic weight of regions to implement the uniformity among the similar image patches by utilizing the prior information.
- Furthermore, we optimize the salient regions map by applying the guided filter, which removes the artifacts and further improves the qualitative as well as the quantitative results.

The remaining part of the paper is organized as follows. The current literature about the SRD is discussed in Section 2. In Section 3, different stages of our method like dictionary construction, saliency detection, and refinement processes are discussed in detail. The comparison of our model with the seven most recent methods is given in Section 4. The conclusion of our method is summarized in Section 5.

## 2. Related Work

Several computational methods are proposed for SRD. The majority of the preceding schemes are appearance-based models, these models mainly depend upon the global or local contrast for their saliency map computation.

### 2.1. Dictionary Learning-Based SRD

The dictionary-based approaches [2,12,13,14,15] facilitate learning multifaceted labeling procedures and represent the image in a space where it can be easily processed. In [12], the basis vector is computed on the belief that the repeatedly activated bases contain less energy as compared to the rare bases. This model works selectively because the unpredicted bases are selected as salient clues. A dictionary for an image patch is constructed from a depository of natural images in [6]. Then, the sparse representation is utilized to find the contrast between each image patch. Shen et al. [13] optimize the objective of feature transformation and low-rank decomposition for training the dictionary. However, these methods manually trained their dictionaries using the top-down way. In [1,14], the authors constructed the dictionary by only utilizing the center-surrounded patches without any training. However, the saliency results are not satisfactory because the inner-region of the salient object is not detected properly. In recent dictionary-based method [8], the author utilized the boundary information to extract the background dictionary. The saliency computed through this background dictionary is not clear because only the boundary information for background dictionary construction is insufficient. Currently, some methods engaged the center-remaining strategy [16], while other used the more background regions [17] to construct their background dictionary. However, most of the time, the background templates contain limited information that leads to incorrect SRD.

### 2.2. Sparse Representation-Based SRD

The image boundary is always standing out as a part of the background. So, it can be very helpful in constructing the background template set [8,9,10]. The authors computed the sparse representation error through this background template set. However, the computed results are not significant when the salient object is touching the image boundary. The center-surrounded strategy is helpful in detecting, so the authors in [16] engaged the center-remaining procedure to extract the dictionary. Then, the sparse reconstruction error is calculated through this dictionary. The computed saliency results averaged and improved through a multi-label inference process. To enhance the difference between the salient object and the background, a sparse coding-based generative model is discussed in [17]. To capture all information related to the image a superpixel sparse reconstruction-based model is defined in [9]. However, the results generated by these models are not very clear because these methods only utilizing the local image information for SRD. Consequently, all these methods improved their results through an enhancement process, which recovers the lost information.

### 2.3. Global or Local Measures-Based SRD

The previously designed SRD techniques are broadly divided into two categories, local and global methods. The local methods compute the saliency by the rarity of neighbors or surrounded regions. While the global methods extract saliency using the uniqueness of features over the entire scene. In [14], the authors computed the saliency as the center-remaining difference of many features. Graph-based SRD method [18] exploits the rarity of different local features to compute the saliency map. A fuzzy growing approach is utilized to compute the saliency with the contrast of neighboring superpixels [19]. Ming Lin et al. [20] proposed the saliency of superpixels by incorporating the global features, namely spatial distribution and uniqueness. They used the PCA method to incorporate color and pattern distinctness to find the SRD. In [7], the authors computed the saliency by the global contrast between the image patches and their spatial position. They performed sampling based on the conventional three-color cues maps and PCA to extract the main features of the image patches. To extract a saliency map with high resolution that is dependent on color contrast, a Histogram Contrast (HC) method is defined in [21]. In [22], a non-local histogram approach is engaged to improve the efficiency of the method, and a smoothing procedure is applied to get rid of quantization artifacts. However, these proposed techniques are only suitable for simple natural images and lose their accuracy for highly patterned and textured images.

### 2.4. Multiple Feature-Based SRD

The existing approaches for SRD are mainly focusing on the color features while ignoring the other features like texture, structure, and the orientation. Therefore, these types of methods are not successful when dealing with an image that contains rich textural features. Many approaches for SRD use the RGB color model and few of them depending upon LAB or $YC_{b}C_{r}$ color space for their result calculation. The authors consider the near-infrared region with the RGB color model for SRD [23], as tthe near-infrared region provides more clues for recognition and categorization than the RGB color model. SRD using sparsity-based and graph-based models is proposed in [24]; the authors combine the multi-features of colors with sparse representation model to compute the saliency. A method for SRD by combining multiple features of color distribution and contrast is proposed in [25], the authors exploited a multi-features color difference measure, a multi-features color distribution measure, and a multi-features salient object measure to compute the saliency. To exploit the multi-features constructing through image manifold of the different feature, a multi-feature enhancement procedure is discussed in [16]. However, these methods add some high contrast pixels with the salient object that lead to insignificant detection.

### 2.5. Foreground or Background-Based SRD

The discriminative schemes are also very important because these schemes help in enhancing the contrast between the background and foreground regions for SRD [25]. A number of discriminative strategies based models have appeared in current years. Shuang Li et al., [26] suggested that the saliency of a region is computed by the distance from the most assured background and foreground seeds. Hongyang Li et al., [27] proposed that the saliency of an object is estimated through propagating the cues extracted mainly from the certain object regions and background. The graph-based methods can capture more grouping features in the scene with the graph likeness. Graph similarity typically controls the performance of a graph-based method [11]. Some of them used the semi-supervised learning to approximate the similarities by incorporating local-grouping features deduced from the whole image. The foreground represents appearance consistency and uniformity, while the background many times reveals global or local connectivity with each of the four image boundaries [28]. In [17], a two-stage saliency scheme is defined which is based on relevance to the given query. After that, they used the graph-based manifold ranking procedure to rank the foreground and background cues. However, if the contrast is far from being between the foreground and the background, the computed saliency results are not accurate. Furthermore, it is very difficult to choose the position and the number of salient queries because these cues are generated through the random walks on the graphs, especially for the images that contain, unlike salient objects.

### 2.6. Deep Convolutional Neural Networks-Based SRD

Since Deep Convolutional Neural Networks (CNN)-based methods [29,30,31] are engaged for SRD, tremendous progress has been achieved because of the availability of large visual datasets and GPU computing resources. The development of deeper and larger DCNNs [29,30,31] that could automatically learn more and more powerful feature representations with multiple levels of abstraction from big data. Significant progress has been made in the past few years to boost the accuracy levels of SRD [29,30,31], but existing solutions often rely on computationally expensive feature representation and learning approaches, which are too slow for numerous applications. In addition to the opportunities they offer, the large visual datasets also lead to the challenge of scaling up while retaining the efficiency of learning approaches and representations for both handcrafted and deeply learned features. In addition, given sufficient amount of annotated visual data, some existing features, especially DCNN features [29,30,31], have been shown to yield high accuracy for visual recognition. However, there are many applications where only limited amounts of annotated training data can be available or collecting labeled training data is too expensive. Such applications impose great challenges to many existing features.

## 3. The Proposed Salient Regions Detection Approach

**Figure 1:**
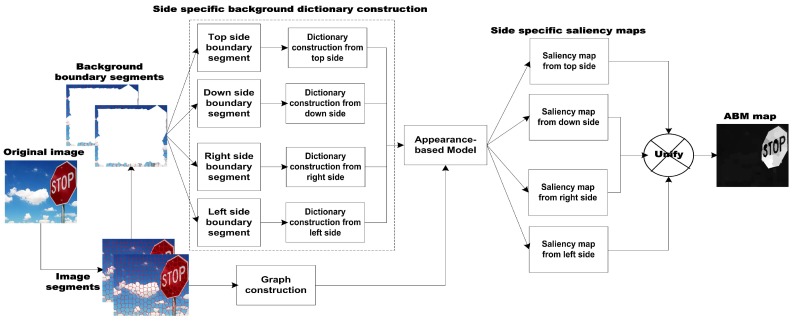
The pipeline of proposed salient region detection model.

In this section, we present the particulars of our proposed approach in detail. In the first stage, we employ the Appearance-Based Model (ABM) to compute the coarse dense salient region map. In the second stage, we engage the Regression-Based Model (RBM) to enhance the discrimination between the foreground and background cues, respectively. Each of the individual stages of the proposed salient region detection method is illustrated in Figure 1.

### 3.1. The Visual Feature Extraction

To encode and accomplish better structural information regarding the image, we first segment the input image into superpixels by utilizing the Simple Linear Iterative Clustering (SLIC) mechanism [32]. SLIC adapts a k-means clustering approach to efficiently generate superpixels. Despite its simplicity, SLIC adheres to boundaries as well as or better than previous methods. At the same time, it is faster and more memory efficient, improves segmentation performance, and is straightforward to extend to super voxel generation. SLIC algorithm group pixels into perceptually meaningful atomic regions which can be used to replace the rigid structure of the pixel grid. SLIC captures image redundancy, provide a convenient primitive from which to compute image features, and greatly reduce the complexity of subsequent image processing tasks. Superpixels present a better method for obtaining the features of an image. As discussed in [6], the conventional color model is supportive for SRD because the colors surround the major part of the image. To capture more information relating to the image, we used the mean of the RGB and CIE Lab color space to represent a superpixel as $Z=[R\;G\;B\;L\;a\;b\;x\;y\;g_{i}\;u_{i}]$, where R, G, B, and L, a, b express the values of RGB color model and CIE Lab color space, respectively while the *x* and *y* express the coordinates of the pixels. Whereas $u_{i}$ is used to indicate the density of edges. Where $g_{i}$ is used to highlight the salient object part through the following Gaussian function:

(1) $$\begin{array}{c} g_{i}=\exp\left[-\left(\frac{x_{i}-x_{c}}{2\sigma_{x}^{2}}-\frac{y_{i}-y_{c}}{2\sigma_{y}^{2}}\right)\right] \end{array}$$

where, $\sigma_{x}=x_{c}$ and $\sigma_{y}=y_{c}$ are the image center co-ordinates, $x_{i}$ and $y_{i}$ indicate the superpixel co-ordinates, $s_{i}$ and $s_{j}$ are the *i*th and *j*th superpixels of the image. Sometimes due to less contrast or same color of the foreground and the background part is mistakenly considered as foreground. To overcome this issue, our focus is salient object instead of image center. To achieve this objective, we calculate salient object center using the following equation:

(2) $$\begin{array}{c} s_{c}=\left\{\begin{array}{c} x_{c}=\sum_{i,j=1}^{n}\frac{s_{i}}{\sum_{j=1}^{n}s_{j}}x_{i}\\ y_{c}=\sum_{i,j=1}^{n}\frac{s_{i}}{\sum_{j=1}^{n}s_{j}}y_{i} \end{array}\right. \end{array}$$

**Figure 2:**
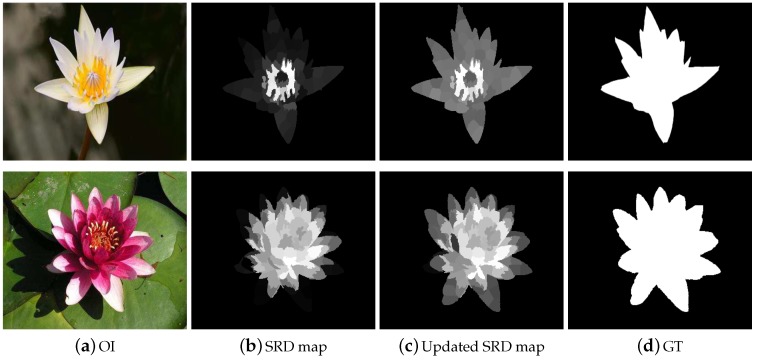
The need for visual features for extracting a good saliency result is obvious from the depicted results. It is worth noting that the results in the second column are comparably less significant and missing a lot of real image information.

Subsequently, the image is presented as $Z=\left[z_{1},z_{2},z_{3},\ldots ,z_{n}\right]\epsilon R^{D\times N}$, where *N* and *D* are the number of segments and features dimensions of the image, respectively. As a result, the calculated saliency maps with textural information have more effective representation as shown in Figure 2b,c, respectively.

### 3.2. Heuristic Background Dictionary

In current SRD schemes, the background contrast, background prior, and boundary information is used to compute their SRD map. Following the previous assumptions, we also assembled a part of the background and boundary clues and named it as a Heuristic Background Dictionary (HBD). Since constructing this HBD, we also used the idea of center-remaining difference to capture high contrast around the salient objects near the center of the image. The HBD has persuasive results for simple natural images, however, for complex natural images, the resultant map contains a large amount of background noise. When the foreground region and background regions are implicated, and the contrast is much smaller, the HBD is less helpful for finding the foreground region. Consequently, when the background is complex it is difficult for ABM to train the HBD which is not capable of extracting complete information from the background, as a result, the salient region map contains background noises. To achieve improved SRD results, we accumulate the accurate background and boundary clues as for the dictionary bases. We computed the value of a segment *i* through the following expression:

(3) $$\begin{array}{c} U_{seg(i)}=\frac{\sum_{L=\{right,left,top,down\}}S_{i,L}.\varphi \left(seg\left(i\right)\notin seg_{L}\right)}{\sum_{L=\{right,left,top,down\}}\varphi \left(seg\left(i\right)\notin seg_{L}\right)} \end{array}$$

where, φ(.) and $seg_{L}$ represent the indicator function boundary segment set, respectively. According to [33,34], the different dataset contains the different size of the salient part and the largest salient object contains the 35% of the image. In a 15-pixel wide narrow border region, 98% belongs to the background [35]. Using this information, we selected the 30% of background pixels for constructing the dictionary. We used the dictionary-learning procedure to avoid the redundant sampling and computational problem in which the background samples are directly utilized as dictionary bases. This training procedure computes more compact heuristic background dictionary $T=\left[t_{1},t_{2},t_{3},\ldots ,t_{n}\right]\in Q^{p\times n}$. We use the following function to compute HBD as:

(4) $$\begin{array}{c} J_{T,E}=\arg\min_{T,E}\left\{{∥\Upsilon -TE∥}_{F}^{2}+\nu {∥E∥}_{1}\right\}\;\;\;s.\;t.\;\;\;t_{j}^{⊤}t_{j}=1,\forall j \end{array}$$

where, $\Upsilon \in R^{p\times n}$ used to signify the background segments sets, E is Representation-Coefficient Matrix (RCM) of Υ based on T, while ν is used to balance the $\ell_{F}-norm$ and $\ell_{1}-norm$ terms. The Equation (4) represents a joint-optimization function of *T* and *E*. Firstly, the *T* is initialized and fixed after that *E* is solved using [36] as it becomes a standard optimization problem. Then, we update *T* by fixing *E* through the Lagrange multiplier. This procedure is iterated till the values of $I_{T,E}$ are close enough and at that time, we are able to obtain a more reconstructive dictionary.

**Figure 3:**
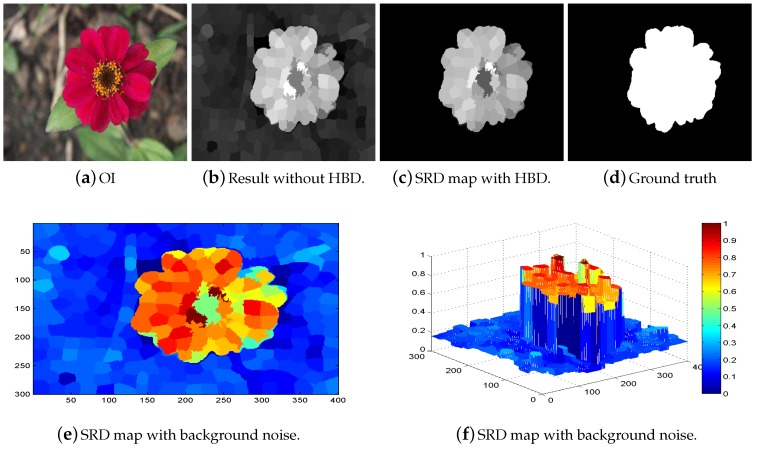

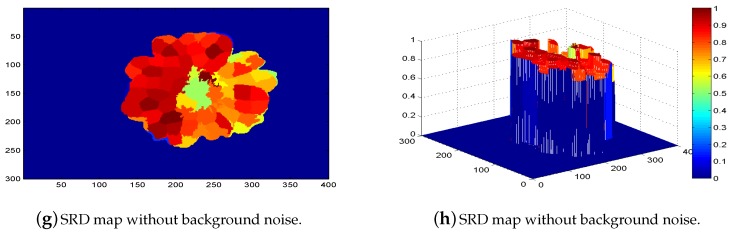
The effectiveness of the heuristic background dictionary for highly precise and exact salient object maps extraction.

The compact appearance frameworks construct their background coefficient matrix which detains all of the fundamental characteristics of the background part, however, it is very sensitive to background noises. The dense appearance models provide more meaningful and basic descriptions of the background region as compared to the foreground region. For messy and complicated scenes, the ABM is less useful in computing the salient objects. So, we use the background contrast from four sides of the image boundary and designed four HBDs. Suppose, if the HBD cannot capture all of the information from one side of the image it will definitely collect some background information from the other sides. The salient objects are more accurately captured if we apply the clues and seed extracted from the four sides of the image. The proposed model HBD is designed to handle these issues. In view of the fact that the distinctive border of the image may possibly enclose a component of the salient object parts, the HBD is very effective and capable of appreciably eradicating these regions of the image that are considered as background noises as revealed in Figure 3. Subsequently, the left behind a set of superpixels is preferred as HBDs, which contain additional stable and consistent background information.

### 3.3. Appearance-Based Salient Region Detection

Superpixels appearance based saliency computation is the most important step of our model. The image boundary superpixel contains very important information which can be engaged to obtain the saliency maps. The methods based on a background dictionary [9,10,11] have convincing results whenever the salient objects pop out closer to the center part of the scene. However, when the salient objects significantly touch the image boundary and parts of them are wrongly considered as background. However, our designed HBD $T=[t_{1},t_{2},\ldots ,t_{m}]$ has D-dimensional cues of boundary and 35% of background segments. We apply this reconstructive background dictionary to remove the background noise and to compute ABM saliency map. The classical SRD method [7,8] computes the dissimilarity between the coefficient of segment *i* as follows:

(5) $$\begin{array}{c} \alpha_{i}=V_{T}^{⊤}\left(z_{i}-\bar{z}\right) \end{array}$$

where, $\bar{z}=\sum_{i=1}^{n}z_{i}$ is the mean feature of *Z* and the eigenvalue and eigenvector is calculated via the normalized covariance matrix of *T*, $V_{T}=\left[v_{1},v_{2},v_{3},\ldots v_{\overset{´}{E}}\right]$. Then, the largest eigenvalues are selected to form the PCA bases for the reconstructive background dictionary. The corresponding saliency of segment *i* can be calculated using the following expression:

(6) $$\begin{array}{c} e_{i}={∥z_{i}-\left(V_{T}\alpha_{i}+\bar{z}\right)∥}_{2}^{2} \end{array}$$

We believe that the dense representation is more expressive to the background features, and it is more sensitive towards the noise. In general, the background part of the image is comparably uniform, sparse, on the contrary, the foreground part is comparably lesser and dense. The key motive for selecting the PCA framework is this when the salient objects are located at the image boundaries. In these typical cases, the background is the main ingredient. So, PCA can easily detect the foreground and filter outs the background. The PCA only deals with simple natural images, however, for complex natural images the resultant map contains a large amount of foreground noise. For cluttered images, the ABM is less effective in measuring salient regions. Dense appearance models, data points through a multivariate Gaussian distribution in feature space, and therefore, it is very difficult to detain multiple scattered patterns particularly when the number of examples is limited. To accomplish better performance of salient region detection, we need to accumulate more correct background information as reconstructive background dictionary bases. We use the background contrast from four sides of the image boundary and designed four HBDs. By utilizing the reconstructive background coefficient set from the top side, we compute the dense representation co-efficient of segment *i* as follows:

(7) $$\begin{array}{c} \alpha_{i,right}=V_{S_{i,right}}^{⊤}\left(z_{i}-\bar{z}\right) \end{array}$$

The saliency value of each segment is proportional to the dense representation. The dense representation of segment *i* using the topside dictionary can be calculated using the following expression:

(8) $$\begin{array}{c} e_{i,right}={∥z_{i}-\left(V_{S_{i,right}}\alpha_{i,right}+\bar{z}\right)∥}_{2}^{2} \end{array}$$

**Figure 4:**
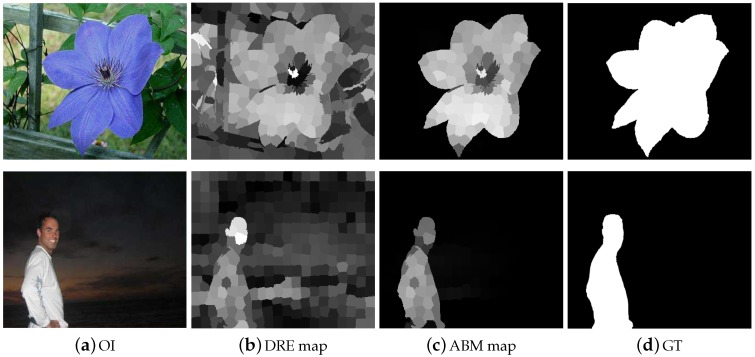
The validity of obtaining a background coefficient matrix is noticeable from the demonstrated results. The results are arranged as OI, the dense representation error map, ABM map, and the GT.

Particularly, the coarse salient region map of each superpixel *z* in a region *r* is extracted as follows:

(9) $$\begin{array}{c} S_{i,top}^{ABM}=\frac{1}{\left|r\right|}\sum_{z_{i}\in r}\left(1-d_{i}\right)\times e_{i,top} \end{array}$$

where $d_{i}$ is the Euclidean distance of the superpixel $x_{i}$ from the center part of the image, and |r| express the numbers of superpixels in *r*. At the end, we normalize $S_{i,right}^{ABM}$, i=1,…,n in the range [0,1] to generate the coarse salient region map from topside. Then the saliency maps are generated from remaining sides likewise and combined to generate $S^{ABM}$ salient region map as depicted in Figure 4. Commonly, the salient part of the image is compact and restricted in a small part which is similar in appearance and consistency, whilst the background part is spread over the whole scene with the same pattern and uniformity. Thus, the superpixels in their correspondences sharing their geometrical appearance information and also their saliency scores. This thing specifies that the average remaining in a superpixel is equal to the saliency values in each region. Additionally, this averaging framework is designed to get rid of the most basic issue in saliency like: a number of small segments having higher contrast values are described through high saliency values sometimes, so the overall saliency of the entire salient object is comparably decreased.

### 3.4. Saliency Enhancement through a Regression-Based Model

We compute a graph G=(V,E), where *V* is set of superpixels and *E* represents the boundary edges of the image. In [16,24,25], the following function is used to determine the saliency of all the superpixels as:

(10) $$\begin{array}{c} F=\arg\min_{F}\left(\sum_{i,j=1}^{n}\underset{Smoothness}{\underset{︸}{w_{ij}{\left(p_{i}-q_{j}\right)}^{2}}}+\beta \sum_{i=1}^{n}\underset{Fitting}{\underset{︸}{{\left(F_{i}-r_{i}\right)}^{2}}}\right) \end{array}$$

where $r_{i}$ is the ranking value for *i*th superpixel, $p_{i}=\frac{F_{i}}{\sqrt{g_{ii}}}$ is saliency of *i*th superpixel, and $q_{j}=\frac{F_{j}}{\sqrt{g_{jj}}}$ is the saliency of *j*th superpixel. $W={\left(w_{ij}\right)}_{n\times n}$ is the weight among two superpixels in the CIE LAB color space and is defined as follows:

(11) $$\begin{array}{c} w_{ij}=\exp-\frac{∥c_{i}-c_{j}∥}{2\sigma_{w}^{2}} \end{array}$$

while $c_{i}$ and $c_{j}$ represent mean of superpixels *i* and *j* in a color model, respectively. Here $\sigma_{w}$ is engage to balance the color weight. Equation (10) illustrates the energy function, the first expression in the Equation (10) is smoothness constraint while the second part is fitting constraint. Therefore, the ranking values of unranked data are computed by solving the above function as:

(12) $$\begin{array}{c} C={\left(D-\varphi W\right)}^{-1} \end{array}$$

where, $D=diag\{d_{11},\ldots ,d_{nn}\}$, and $d_{ii}=\sum_{j}W_{ij}$ are degree matrix and weight matrix, respectively. While the parameter φ keeps a balance between the smoothness constraint and the fitting constraint. Basically, the optimized graph affinities are described through the inverse matrix *C*, these graph-affinities are extracted from the prearranged data signified as a graph through semi-supervised learning without integrating. It also specifies the overall weight between two connected superpixels and extracts their grouping information for SRD. We suppose that an image contains *k* types of features, so weight matrix and degree matrix are computed for *k* features as: $W_{k}={\left(w_{ij}^{k}\right)}_{n\times n}$, and $D_{k}={\left(d_{ij}^{k}\right)}_{n\times n}$. In our designed cost function, we take two n×1 vectors *U* and *V*, which are attained from the previous saliency results by normalizing in the interval of 0∼1. After that, we introduce two diagonal matrices $v=\left[v_{ii}\right]=diag\left(V\right)$ and $u=\left[u_{ii}\right]=diag\left(U\right)$. To combine numerous features in a single salient region map containing the smoother foreground and suppress background, we define our novel pairwise potential model as:

(13) $$\begin{array}{c} F_{l}=\arg\min_{F_{l},l=1..,k}\sum_{l=1}^{k}\left(\underset{Smoothness}{\underset{︸}{\lambda \sum_{i=1}^{n}\sum_{j=1}^{m}w_{ij}^{l}{\left(F_{i}^{l}-F_{j}^{l}\right)}^{2}}}+\underset{Foreground}{\underset{︸}{\sum_{i=1}^{n}u_{ii}^{l}{\left(F_{i}^{l}-1\right)}^{2}}}+\underset{Background}{\underset{︸}{\sum_{i=1}^{n}\left(1-v_{ii}^{l}\right){\left(F_{i}^{l}\right)}^{2}}}\right) \end{array}$$

where, $F_{i}$ and $F_{j}$ are saliency values of segment *i* and segment *j*, respectively. While the λ is a balancing parameter. The first term on the right-hand side in energy function is the smoothness constraint. For a good saliency map the salient object should be even and smooth. The second term is used here to assign higher values to the foreground region. We employ this term for multi-features foreground computation and highlighting the foreground part. The last defined constraint is background constraint which assigns less weight to the background regions and also helps in creating well-defined boundaries of the salient objects. Previously designed methods are dependent on the color information for computing their saliency. However, the computed images lose their accuracy when the salient objects are pattern objects. To fully capture the salient objects, we combine the boundary, texture, geometry and spatial information to obtain our saliency results. The mean of color features are obtained from the superpixels and utilized after normalizing it. While the textural features like HOG and LBP feature are also extracted from the superpixels but after normalizing their histogram. The sum of texture and color discontinuities is computed through gradient *G* and utilized it as the boundary information. All of the above features are utilized to compute the weights of superpixels as:

(14) $$\begin{array}{c} w_{ij}^{l}=\exp-\left(\sum_{l=1}^{k}\left(\frac{∥c_{i}^{l}-c_{j}^{l}∥}{\sigma_{w}^{2}}\right)+\beta \sum_{l=1}^{k}d_{L}\left(L_{i}^{l},L_{j}^{l}\right)+\gamma \sum_{l=1}^{k}d_{H}\left(H_{i}^{l},H_{j}^{l}\right)\right) \end{array}$$

where, the β and γ are used to control the weights between the superpixels. Here, we assign highest weight to the color parameter because it is more reliable than other features. We take the value of k=2, because in this framework we are only dealing with two features. After putting the value of *k* this optimization function can be written as:

(15) $$\begin{array}{c} \begin{array}{c} F_{1},F_{2}=\arg\min_{F_{1},F_{2}}\frac{1}{2}(\lambda F_{1}^{⊤}\left(D_{1}-W_{1}\right)F_{1}+\frac{1}{2}u{\left(F_{1}-D_{1}^{-1}\right)}^{⊤}D_{1}\left(F_{1}-D_{1}^{-1}\right)+\lambda F_{2}^{⊤}\left(D_{2}-W_{2}\right)F_{2}+\\ \frac{1}{2}u{\left(F_{2}-D_{2}^{-1}\right)}^{⊤}D_{2}\left(F_{2}-D_{2}^{-1}\right)+\frac{1}{2}F_{1}\left(1-v\right)F_{1}^{⊤}) \end{array} \end{array}$$

We took the value of k=2 to compute the optimal solution of this energy function. We take the derivative of this function with respect to $F_{1}$ and $F_{2}$ and putting it equal zero. Then we obtained the following expression as:

(16) $$\begin{array}{c} E_{1}=2\lambda \left(D_{1}-W_{1}\right)+uD_{1}+\left(I-v\right) \end{array}$$

(17) $$\begin{array}{c} E_{2}=2\lambda \left(D_{2}-W_{2}\right)+uD_{2}+\left(I-v\right) \end{array}$$

**Figure 5:**
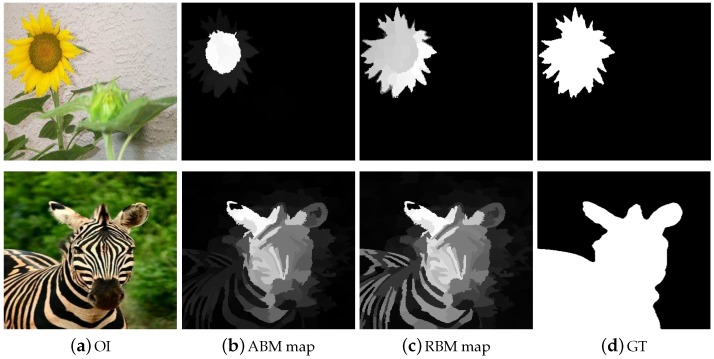
Some examples demonstrating the difference between single and multi-level cues integration step. The results are arranged as OI, salient region map with single feature integration, and the saliency map extracted through multi-label features incorporation.

Motivated from [6], which observed the paired advantages of Lab and RGB color models for salient region detection, we engaged two types of visual information like $E_{1}$ and $E_{2}$ to extract our results. After that, we take the average of the salient region maps and normalize the computed result between the range [0,1] to obtain the final saliency region map. Figure 5 demonstrates the computed results through the proposed model with single and multi-featured.

**Figure 6:**
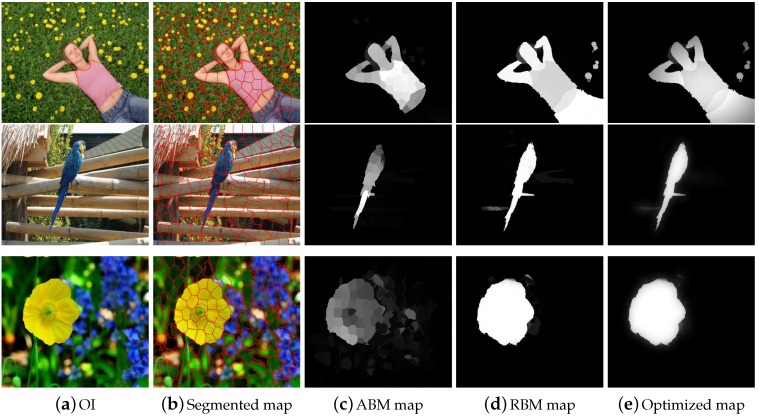
We individually compare the salient region map of each stage of the proposed method by using ASD database [38]. The results are organized as OI, the segmented image, ABM salient region map, enhanced salient region map through RBM, and the final salient region map.

**Figure 7:**
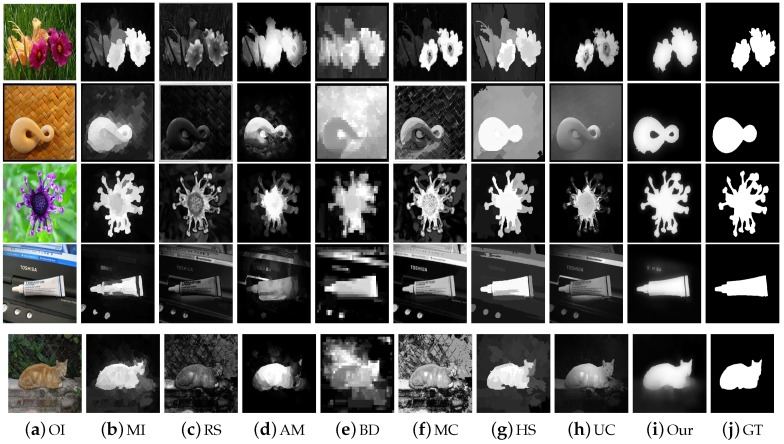
Visual comparison of our scheme with some recent approaches using the ASD database. The SRD results are arranged as OI, MI, RS, AM, BD, MC, HS, UC, our scheme, and the GT. We can note that the SRD maps of our proposed scheme are very close to the GT.

Instinctively, a region with higher contrast in representation to the neighboring elements always receives high saliency scores. However, the proposed multi-feature inference mechanism not only processes the salient regions of the image depending upon their degree of relevance but also assigns higher saliency scores computed from multi-features spaces. This property effects in highlighting the salient object parts more uniformly and suppressing the background regions. We can note that the ABM is more robust in dealing with the salient object at the image boundary. However, for complex natural images, the resultant map contains a large amount of foreground noise. The RBM is more efficient in dealing with the complex background but loses its efficiency when the objects are at the boundary of the image. Consequently, both the RBM and ABM are essential for computing a good salient region map as shown in Figure 6. In very complex background images, sometimes, background pixels included in the results, we can see artifacts in the computed maps due to the pre-processing. So, in order to remove these artifacts and background pixels, we engage the guided filter [37]. The guided filter produces the background and artifacts free smooth result as revealed in Figure 7.

## 4. Experimental Results

We analyzed and investigate our model on the five largest benchmark datasets against the seven state-of-the-art methods. For performance assessment, four evaluation measures are selected to completely analyze the proposed algorithm against seven preceding schemes. In the next section, we discuss the details of the selected benchmark datasets for performance evaluations.

### 4.1. Benchmark Datasets

To analyze the computed saliency results, there are many databases available that differ from one and another in size, number objects, and background. We employ a different database to assess and analyze the performance of our proposed algorithm. We assess our salient region detection model on five different standard databases that are: (1) ASD [38], (2) ECSSD [39], (3) DUT-OMRON [28], (4) SED2 [40], and (5) MSRA [41]. We prefer these databases for the following reasons: (1) background nature, (2) complexity level, (3) a large number of images, (4) the different number of objects in a scene, and (5) potential benchmark databases. Firstly, we test the performance of the proposed model in the ASD database. The images in this database have a large variety in the background structure like a simple, smooth, complex, and multifaceted nature. The ASD database contains 1000 images with pixel-wise annotated ground truths. The purpose to include SED2 databases is to assess the performance of our model with an image contains multiple objects. Lastly, we analyze our model over Extended Complex Scene Saliency Data-set (ECSSD), which contains 1000 images that are semantically meaningful, however, having complex and natural images.

### 4.2. Preceding Methods Selected for Comparison

Our SRD model is compared against seven state-of-the-art models. We first visually and then graphically compare to check and validate our framework. The schemes we compare with our method are chosen due to the following four reasons: (1) recency, (2) citations, (3) computation complexity, and (4) variety. These models are: AM [29], BD [42], RS [43], MC [44], MI [30], HS [39], and UC [31]. The source codes of some of the above-defined approaches are easily accessible for public. While other we obtained from the saliency results generated by Cheng et al. [34]. Only a few of the source codes are directly downloaded from the author’s web, therefore, we utilized their source codes to extract the saliency results for comparison purpose.

### 4.3. Evaluation Metrics

Numerous techniques are applied to evaluate the concurrence between the obtained results and the GT. Before computing the evaluation metrics, the produced salient region maps should be changed in binary form to estimate the generated map. We also apply the adaptive threshold as discussed in [34], the thresholding is used to get the binary mask of salient region map *S*, that is calculated as:

(18) $$\begin{array}{c} T_{h}=\frac{1}{w\times h}\sum_{a=1}^{h}\sum_{b=1}^{w}S\left(a,b\right) \end{array}$$

whereas, *w* and *h* represent the height and width of saliency map, respectively.

#### 4.3.1. Precision-Recall

The saliency map *S* is converted to the binary-mask *M* using the given ground truth *T*. The PR-curve is computed using this expression:

(19) $$\begin{array}{c} Precision=\frac{\left|M⋂T\right|}{\left|M\right|},Recall=\frac{\left|T⋂M\right|}{\left|T\right|} \end{array}$$

#### 4.3.2. F-Score

F-score is calculated using the Precision-Recall, the evaluation of the SRD is not complete without F-score. The F-score is computed using the following expression:

(20) $$\begin{array}{c} F_{\nu }=\frac{(1+\nu^{2})\times Precision\times Recall}{\nu^{2}\times \left(Precision+Recall\right)} \end{array}$$

All of the compared method take the value of ν=0.3. So, we have take the value of ν=0.3 for a fair comparison.

#### 4.3.3. Receiver Operating Characteristics

The ROC-curve is obtained using the binary mask M with a fixed threshold as:

(21) $$\begin{array}{c} TPR=\frac{|\bar{M}⋂T|}{|\bar{M}|},FPR=\frac{|M⋂\bar{T}|}{|\bar{T}|.} \end{array}$$

where, $\bar{T}$ is opposite of *T* and $\bar{M}$ is opposite of *M*. The ROC-curve is obtained through TPR and FPR with changing the value of the fixed threshold.

#### 4.3.4. Mean Absolute Error

To check the worth of SRD maps might have high significance as compared to binary mask. We also applied the MAE between the continuous SRD map *S* and the ground truth *T*, both are normalized in the range [0,1]. The MAE value is defined as:

(22) $$\begin{array}{c} MAE=\frac{1}{w\times h}\sum_{a=1}^{h}\sum_{b=1}^{w}\left|\bar{S}\left(a,b\right)-\bar{T}\left(a,b\right)\right| \end{array}$$

### 4.4. Implementation and Analysis

We visually and graphically analyze the designed algorithm against preceding algorithms. We also assess the performance of the proposed model with different parameters using PR-curves. In the next section, we describe the comparison of our model with existing schemes.

#### 4.4.1. Parameter Settings

**Figure 8:**
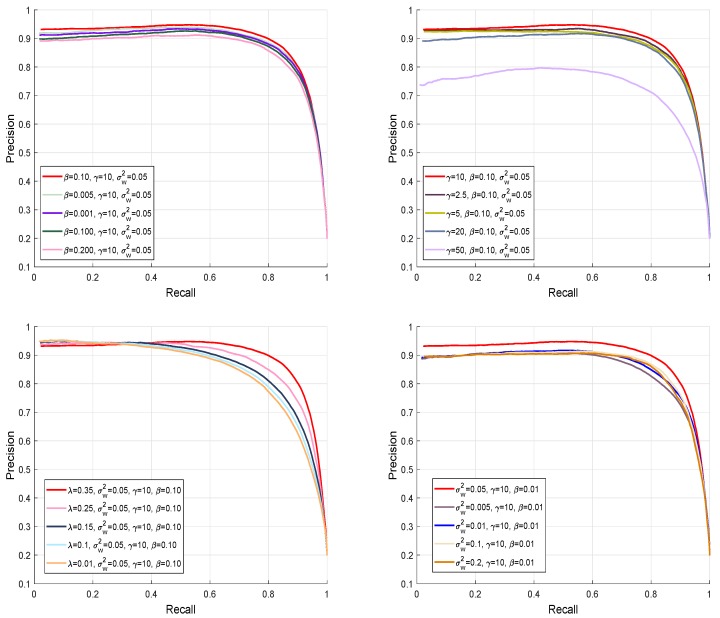
PR-curves to validate our proposed method with different parameters values for the MSRA database. The balancing parameter is tuned at different values to verify the refinement function and their effect on the final SRD map.

The performance of our model is affected by different parameters. When we are comparing the performance of our model, we used the following parameter settings: β=0.10, γ=10, λ=0.35, $\sigma_{w}=0.05$, and N=200, where *N* represents the number of superpixels. Figure 8 demonstrates the effect of these balancing parameters on the performance of our model. We execute simulations 5 times repetitively to avoid any uncertainty due to the arbitrary initialization.

#### 4.4.2. Evaluation of Our Algorithm

**Figure 9:**
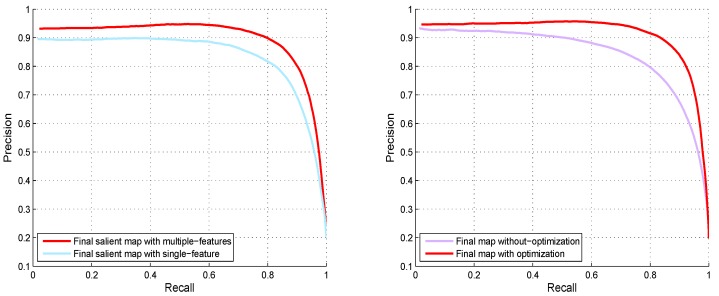
Graphical performance comparison of different stages of our method using PR-curves to validate the single feature, multi-featured, and enhanced results using the MSRA dataset.

In this section, we evaluate different elements of the designed framework and their impact on the performance in detail. The PR-curves with and with the single and multi-features are also demonstrated in Figure 9. We can also see that the final map with the multi-features is little higher than the final map with a single feature. The final map with a single-feature loses some information during pre-processing. We evaluate the proposed method against two most recent SRD schemes: NS [45], and MSC [46] in Table 1. We used the F-measure, AUC, and MAE to check the performance of our model against these two schemes. We notice that our model outperforms than the opponent schemes in selected metrics with higher F-score, AUC and lesser MAE.

#### 4.4.3. ASD Database

**Figure 10:**
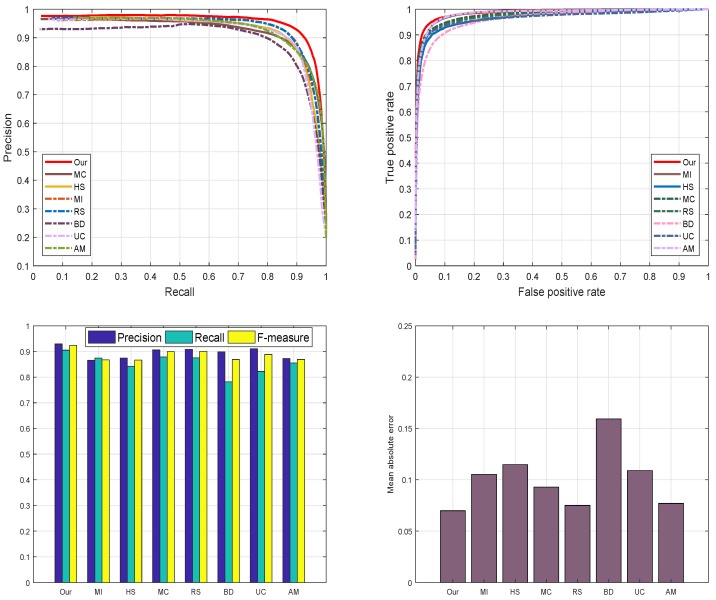
The graphical assessment of our model against seven current approaches AM [29], BD [42], RS [43], MC [44], MI [30], HS [39], UC [31] and our proposed model using the ASD dataset.

We assess the performance of our scheme with previous methods using the ASD dataset as revealed in Figure 10. The reason for selecting the ASD database is to investigate the behavior of our scheme with images having different complexity levels and diversified pattern. We examine and evaluate the proposed method against seven most well-known SRD schemes such as: AM [29], BD [42], RS [43], MC [44], MI [30], HS [39], and UC [31]. We used the ROC-curve, F-measure, PR-curve, and MAE to check the performance of our model. We notice that our model outperforms than the opponent schemes in selected metrics with a higher precision, recall, F-measure, and lesser mean absolute error. The RS [43], HS [39], and MC [44] also achieved good. We considers three latest deep learning-based models for evaluation like [29,30,31]. We can note from the Figure 10 that proposed model obtains similar precisions with most deep-learning methods and suppresses the recalls, so the proposed method yields relatively lower F-measure scores. However, the proposed model is without preparing expensive ground truth annotations for training the model and overall performs comparable with these deep-learning methods. The proposed method is free of computing power and ground truth annotations and can provide simplicity and easy-to-use generality in many practical inexpensive applications. From the results, we observe that our SRD approach is more efficient in highlighting the salient objects as compared to the other recent models.

#### 4.4.4. DUT-OMRON Database

**Figure 11:**
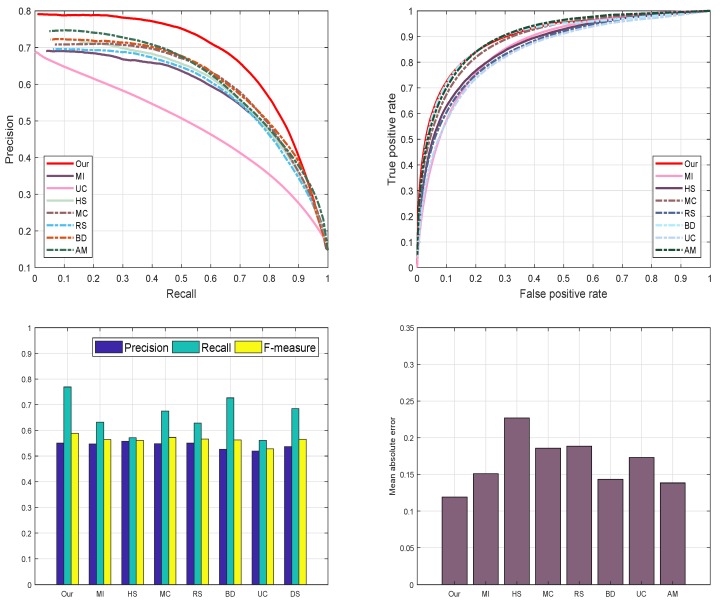
The graphical evaluation of our method with seven current approaches such as AM [29], BD [42], RS [43], MC [44], MI [30], HS [39], UC [31] and our proposed model on the DUT-OMRON database.

We also evaluate the performance of the proposed model on a DUT-OMRON database. The motive for electing DUT-OMRON database is this, it contains a large number of images with different complexity levels of the background. Most probably all SRD approaches utilize this database to analyze their methods, therefore, this database is our first priority to evaluate our proposed approach as shown in Figure 11. We verify the performance of our proposed model graphically using the preprocessing and post-processing results. We choose PR and ROC-curve to assess the performance of our proposed method. The resulting graphs are illustrated in Figure 11. Nevertheless, MC [44], RS [43], and BD [42] also demonstrate persuasive results. We notice from our analysis that our approach is more effective and more efficient in highlighting the salient objects than the other discussed methods.

#### 4.4.5. ECSSD Database

**Figure 12:**
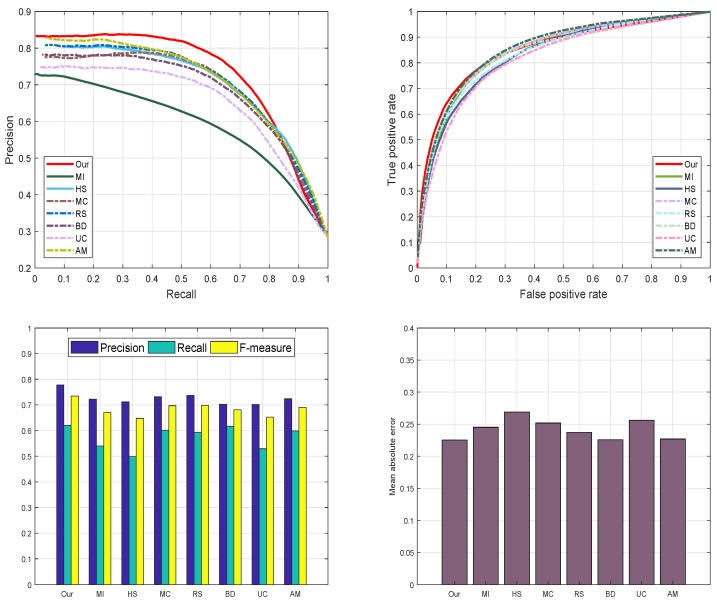
Graphical evaluation of our model using the PR-curve, F-measure, ROC-curve, and MAE with seven most recent models.

Moreover, we as well engaged ECSSD database [39] to assess our mechanism graphically. ECSSD database contains more natural images with a diversified pattern for both foreground and background. The reason for selecting ECSSD database is to investigate the behavior of our scheme with images having different complexity levels and diversified pattern. We examine and evaluate the proposed method against seven most well-known SRD schemes such as: AM [29], BD [42], RS [43], MC [44], MI [30], HS [39], and UC [31] on the ECSSD database to declare the strength of our algorithm. We pick four different criteria which are mainly used in the literature to assess the performance of SRD methods. These criteria are PR-curve, ROC curve, F-score, and MAE to check the performance of our proposed approach. From the series of experiments, we found that our proposed method achieves very good results as compared to above-defined approaches. On the other hand, RS [43], BD [42], and UC [31] as well accomplished fine results on all four SRD metrics. Our approach remains very unswerving in all defined evaluation measures and demonstrates significant performance as shown in Figure 12.

#### 4.4.6. SED2 Data-Set

**Figure 13:**
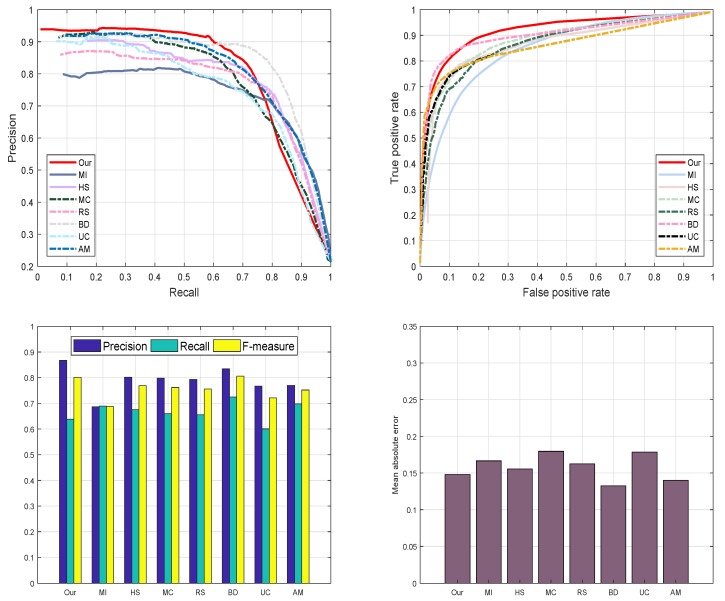
The graphical analysis of our SRD using four different saliency measures with other techniques.

Additionally, we employed SED2 dataset [40] to evaluate and validate the proposed method graphically. The motive for electing SED2 database is to assess the performance of our scheme through an image with two objects. We analyze and compare the proposed method against fourteen most famous state-of-the-art approaches such as: AM [29], BD [42], RS [43], MC [44], MI [30], HS [39], and UC [31] on SED2 database to assure the validity of our algorithm. We choose four different criteria like PR-curve, ROC curve, F-measure, and MAE to estimate the strengths and bounds of our SRD approach. Our SRD model remains very consistent in all the define evaluation measures and shows a remarkable performance as illustrated in Figure 13.

#### 4.4.7. Limitations

**Figure 14:**
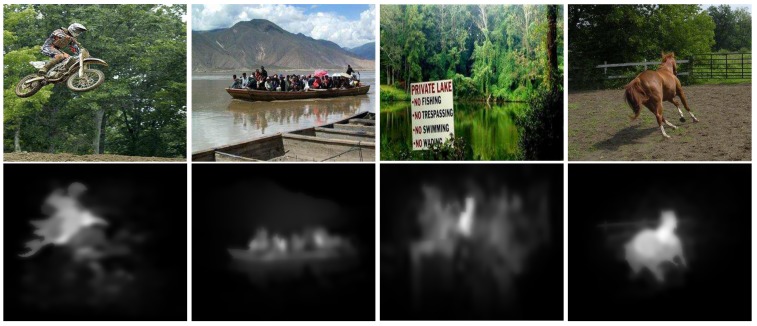
A few cases where our model performance is not very persuasive.

The designed method outperforms against above-discussed state-of-the-art SRD methods with the higher PR values. However, the performance of our scheme is not very acceptable in some cases. These typical cases are shown in Figure 14. The proposed method has not achieved very persuasive results when the color of the foreground is similar to the background; in this situation, the salient object is not salient accurately, some of the background pixels are combined with the obtained results and size of the results do not remain significant.

#### 4.4.8. Execution Time

The execution time/image of the proposed model with some previous methods by using MATLAB implementation using the ECSSD data set is elaborated in Table 2. The running time of all the schemes described in the table is achieved through the machine having the Intel Dual Core i3−2310M, 2.10 GHz CPU, and 4 GB RAM. Our designed framework is robust than the other state-of-the-art SRD methods. Specially, the SLIC [32] consumes 0.16 s almost 50% of the original time.

## 5. Conclusions

In this work, we have introduced a new density-based and regression-based salient regions detection model. To capture the useful structural information, we segmented the image into multiple uniform segments. To obtain more background information and to evenly suppress the background, we constructed side-specific dictionaries. Then, we calculated the more effective contrast-based salient region map using our ABM. To strengthen the generated results, we use RBM to generate the multi-label cues rarity for each segment. To incorporate pre-computed results followed by an optimization method that construct more even, accurate and precise salient regions map. Some previous approaches exploit the single-feature of the background or foreground to produce their saliency results. However, the proposed model infers multi-label color features and demonstrates better performance as compared to the preceding appearance-based learning schemes.

## Figures and Tables

**Table 1:** The performance comparison of our model with recent schemes.

Models	ECSSD			SED2			DUT-OMRON			ASD		
	NS	MSC	Our	NS	MSC	Our	NS	MSC	Our	NS	MSC	Our
**F-score**	0.710	0.713	0.73	0.775	0.791	0.802	0.616	0.60	0.699	0.870	0.92	0.93
**AUC**	0.90	0.89	0.907	0.85	0.859	0.861	0.887	0.883	0.895	0.935	0.952	0.953
**MAE**	0.245	0.229	0.222	0.182	0.155	0.145	0.149	0.126	0.125	0.095	0.080	0.070

**Table 2:** The comparison of our model with seven state-of-the-art techniques for average running time (seconds per image).

Method	Time(s)	Code
AM [29]	0.185	Matlab
BD [42]	0.453	Matlab
MC [44]	0.547	Matlab
MI [30]	0.025	Matlab
UC [31]	0.495	Matlab
RS [43]	0.108	Matlab
HS [39]	25.3	Matlab
Our	0.32	Matlab
